# Long-Term Prognostic Value of the Response to Therapy Assessed by Laboratory and Imaging Findings in Patients with Differentiated Thyroid Cancer

**DOI:** 10.3390/cancers13174338

**Published:** 2021-08-27

**Authors:** Michele Klain, Emilia Zampella, Leandra Piscopo, Fabio Volpe, Mariarosaria Manganelli, Stefania Masone, Leonardo Pace, Domenico Salvatore, Martin Schlumberger, Alberto Cuocolo

**Affiliations:** 1 Department of Advanced Biomedical Sciences, University Federico II, 80131 Naples, Italy; michele.klain@unina.it (M.K.); emilia.zampella@unina.it (E.Z.); leandra.piscopo@unina.it (L.P.); fabio.volpe@unina.it (F.V.); mariarosaria.manganelli@unina.it (M.M.); martin.schlumberger@gustaveroussy.fr (M.S.); 2 Department of Clinical Medicine and Surgery, University Federico II, 80131 Naples, Italy; stefaniamasone@yahoo.it; 3 Department of Medicine, Surgery and Dentistry, University of Salerno, 84084 Salerno, Italy; lpace@unisa.it; 4 Department of Public Health, University Federico II, 80131 Naples, Italy; domenico.salvatore@unina.it

**Keywords:** Differentiated thyroid carcinoma, ^131^I therapy, prognosis

## Abstract

**Simple Summary:**

In patients with differentiated thyroid cancer (DTC), the American Thyroid Association dynamic risk stratification system has been proposed to identify patients at higher risk of recurrence during follow-up. This system is based on a combination of serum thyroglobulin determination and neck ultrasonography obtained 12-months after radioactive iodine (RAI) therapy. Radioiodine diagnostic whole-body scan (WBS) is performed less frequently due to its low sensitivity. In this retrospective study we assessed the long-term predictive value of the response to therapy at 12 months, evaluated by serum thyroglobulin determination and neck ultrasound, and estimated the potential additional impact of diagnostic WBS in patients with DTC treated with surgery and RAI therapy. Our findings could help in the identification of DTC patients at higher risk of recurrence that could benefit from a closer follow-up.

**Abstract:**

This study assessed the long-term predictive value of the response to therapy, evaluated by serum thyroglobulin (Tg) determination and neck ultrasound, and estimated the potential additional impact of diagnostic whole-body scan (WBS) in patients with differentiated thyroid cancer (DTC) treated with surgery and radioactive iodine (RAI) therapy. We retrospectively evaluated 606 DTC patients treated with surgery and RAI. Response to ^131^I therapy at 12 months was assessed by serum Tg measurement, neck ultrasound, and diagnostic WBS. According to American Thyroid Association (ATA) guidelines, patients were classified as having a low, intermediate or high risk of recurrence and at 12 months as having an excellent response (ER) or no-ER. Follow-up was then performed every 6–12 months with serum Tg determination and imaging procedures. With a median follow-up of 105 months (range 10–384), 42 (7%) events requiring further treatments occurred. Twenty-five patients had additional RAI therapy, 11 with structural disease in the thyroid bed, eight in both thyroid bed and neck lymph nodes, four had lung metastases and two had bone metastases. The other 17 patients had additional surgery for nodal disease followed by RAI therapy. The ATA intermediate and high risk of recurrence, post-operative and pre-RAI therapy Tg ≥ 10 ng/mL, and the absence of ER at 12 months were independent predictors of events. Diagnostic WBS at 12 months permitted the identification of only five recurrences among the 219 ER patients according to serum Tg levels and ultrasound. In DTC patients, the response to therapy at 12 months after RAI therapy could rely on serum Tg measurement and neck ultrasound, while diagnostic WBS was not routinely indicated in patients considered in ER.

## 1. Introduction

Although the overall long-term survival of patients with differentiated thyroid cancer (DTC) is excellent, disease recurrence is relatively common in some subsets of DTC patients who can be identified by an accurate risk stratification system [1,2,3]. The American Joint Committee on Cancer/Union for International Cancer Control Tumor Node Metastasis is an initial staging system that predicts mortality [4]. The American Thyroid Association (ATA) initial risk stratification system has been proposed to assess the risk of recurrent or persistent disease in DTC patients [5]. The patients are classified into low, intermediate, and high-risk categories [6]. In addition, a dynamic risk stratification system has been proposed by the ATA based on clinical, biochemical, and imaging data obtained during follow-up [4,5,6]. Radioiodine diagnostic whole-body scanning (WBS) has been largely used in the past for assessment of disease status [7,8]. Currently, WBS scanning is performed less frequently and has been replaced by a combination of serum thyroglobulin (Tg) determination and neck ultrasonography (US). It has been recently reported that the distribution of response to therapy could differ according to the follow-up protocols when diagnostic WBS scanning is considered [9]. However, the prognostic implications of these findings has not yet been fully elucidated. Hence, we assessed the long-term predictive value of the response to therapy at 12 months, evaluated by serum thyroglobulin determination and neck ultrasound, and estimated the potential additional impact of diagnostic WBS in patients with DTC treated with surgery and radioactive iodine (RAI) therapy.

## 2. Materials and Methods

### 2.1. Patients

A total of 712 patients with DTC who were referred to our center between 1992 and 2002 were included. All patients underwent total thyroidectomy, with central and/or lateral neck dissection when appropriate, followed by ^131^I therapy. In particular, 142 patients had total thyroidectomy, 446 had total thyroidectomy and central neck dissection, and 124 patients had total thyroidectomy and lateral neck dissection. Histopathological data were collected, and patients were classified as low, intermediate, or high risk in terms of structural recurrence of disease according to ATA guidelines [5]. Before ^131^I therapy, L-thyroxine treatment was discontinued for 20–30 days until serum TSH levels increased above an arbitrary level of 30 mIU/L. At that time, serum thyroglobulin (Tg) concentration was measured, and ^131^I was administered (range 1110–6808 MBq). Serum Tg levels were determined by immunoradiometric assay (Dynotest Tg, Henning, Berlin, Germany), with a sensitivity of 1 ng/mL up to 1996 and then by chemiluminescence system (Immulite, Diagnostic Products Corp, Los Angeles, CA, USA) with a detection limit of 0.2 ng/mL. According to post-operative Tg values obtained following thyroid hormone withdrawal at the time of ^131^I administration, patients were categorized into two groups: ≥10 ng/mL and <10 ng/mL [10,11]. Five to seven days later, a post-therapy WBS was performed using a dual-head gamma camera (E.CAM, Siemens Medical Systems, Hoffman Estates, IL, USA) equipped with thick crystals and high energy collimators [12]. The patients with evidence of distant metastasis at post-therapy WBS were excluded from the study.

### 2.2. Therapy Response Evaluation

The response to ^131^I therapy at 12 months was assessed with serum Tg measurement obtained on LT4 treatment or following thyroid hormone withdrawal, neck US, and diagnostic WBS. WBS was performed after LT4-withdrawal until the TSH increased above an arbitrary level of 30 mIU/L, and two days after the administration of 185 MBq of ^131^I, using a dual-head gamma camera (E.CAM, Siemens Medical Systems, Hoffman Estates, IL, USA). According to the 2015 ATA guidelines [6,10], definitions of responses to therapy were: (1) excellent response (ER), negative imaging and either Tg < 0.2 ng/mL on LT4 treatment or TSH-stimulated Tg < 1 ng/mL; (2) indeterminate response (IR), non-specific findings on imaging studies or Tg levels on LT4 treatment that are detectable but <1 ng/mL or stimulated Tg levels between 1 and 10 ng/mL or stable or declining titer of Anti-Tg antibodies in the absence of structural disease; (3) biochemical incomplete response (BIR), negative imaging and Tg ≥ 1 ng/mL on LT4 treatment or stimulated Tg ≥ 10 ng/mL or rising titer of anti-Tg antibody; and (4) structural incomplete response (SIR), structural evidence of disease with any level of serum Tg or of anti-Tg antibodies. Response to therapy was assessed considering Tg and US findings and then the results of the diagnostic WBS were analyzed according to the response to therapy. The patients without neck US or diagnostic radioiodine scan at the 12-month evaluation were excluded from the study.

### 2.3. Follow-Up

After the evaluation at 12-months, all patients were followed up with serum Tg level determinations (on L-thyroxine and in some patients off L-thyroxine therapy when withdrawal was performed for a ^131^I WBS) every 6–12 months, and imaging procedures as appropriate. Disease status was recorded at each evaluation. Recurrence of disease was defined by histology or imaging procedures; suspicious nodal abnormalities at neck ultrasonography were confirmed by fine needle aspiration cytology, histology, and the presence of RAI uptake when it corresponded to abnormal findings at neck ultrasonography. All patients with recurrent disease were submitted to further treatments. Patients last known to be alive and progression free were censored at the date of last contact.

### 2.4. Statistical Analysis

Continuous data are expressed as mean ± standard deviation and categorical data as percentage. A student two-sample *t* test and χ^2^ test were used to compare the differences in continuous and categorical variables, respectively. Hazard ratios with 95% confidence intervals (CI) were first calculated by univariate regression analysis. Variables showing a *p* value < 0.05 at univariate analysis were considered statistically significant and were included in the multivariate Cox regression analysis. Statistical analysis was performed with Stata Statistical Software (StataCorp. 2015.: Release 14. StataCorp, College Station, TX, USA).

## 3. Results

Of the total of 712 patients with DTC, 39 with initial distant metastases and 32 without neck US or diagnostic WBS at 12 months were excluded. Of the remaining 641 patients, follow-up was not available in 35 (5%), leaving 606 subjects for the analysis. With a median follow-up of 105 months (range 10–384), 42 (7%) events occurred and required additional treatments. Twenty-five patients had additional ^131^I therapy, 11 with structural disease in the thyroid bed, eight in both thyroid bed and lymph nodes, four for lung metastases and two for bone metastases. The other 17 patients with nodal disease had additional surgery and ^131^I therapy.

Characteristics of the study population at the time of RAI therapy according to events are reported in Table 1. The prevalence of high ATA risk and Tg ≥ 10 ng/mL was higher in patients with events as compared to those without (both *p* < 0.001).

Characteristics of the study population at 12 months evaluation according to events are reported in Table 2. The prevalence of detectable Tg, abnormal US and positive diagnostic WBS was higher in patients with events as compared to those without. According to Tg level and US, 219 patients were classified as ER and 387 as no-ER. Among no-ER patients, 182 were classified as IR, 203 as BIR and 2 as SIR for lymph node metastases confirmed by FNA.

Individual characteristics of 42 patients with events at the time of recurrence are depicted in Table 3.

### Predictors of Events

**Figure 1:**
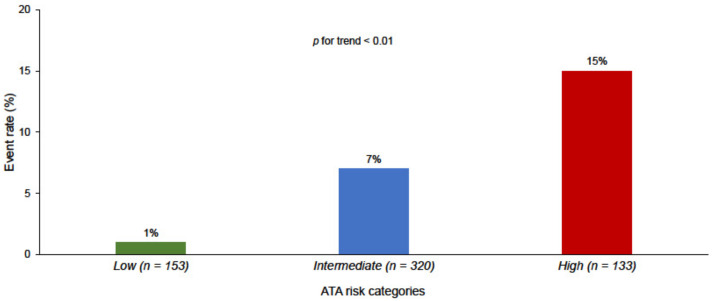
Event rate according to ATA risk categories.

The rate of events was higher in the ATA high risk patients compared to intermediate and low risk patients (*p* for trend < 0.01) (Figure 1). However, the 198 patients with pre-therapy Tg ≥ 10 ng/mL had a higher rate of recurrence as compared to the 408 patients with lower Tg values (14% vs. 3%, *p* < 0.001). Similarly, the rate of structural recurrence was higher in no-ER patients as compared to ER (9% vs. 2%, *p* < 0.01).

**Figure 2:**
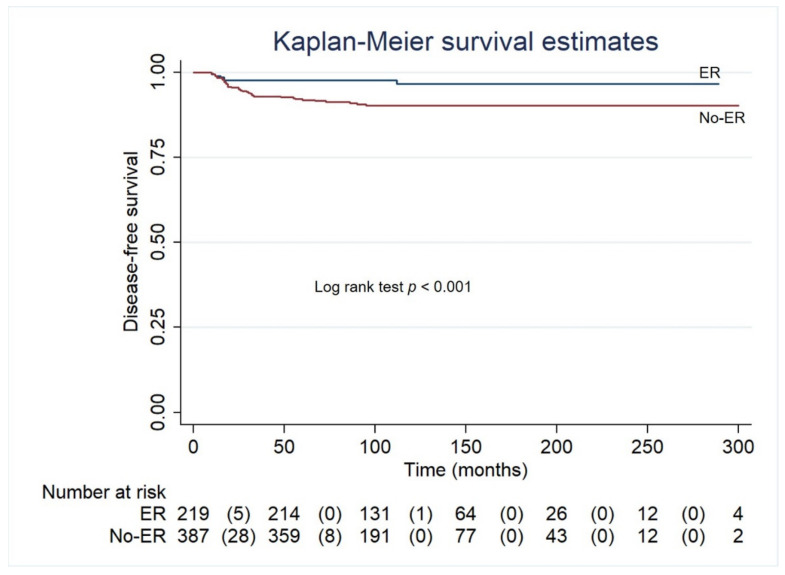
Disease-free survival by Kaplan-Meier in patients with ER and with no-ER at 12 months after RAI therapy. ER (excellent response).

Univariable and multivariable predictors of recurrence are reported in Table 4. ATA risk categories, pre therapy Tg ≥ 10 ng/mL, response to therapy evaluated with serum Tg and neck US at 12 months, were predictors of events at both univariable and multivariable analyses. At Kaplan Meier analysis, disease free survival was lower in patients with no-ER at 12 months after RAI therapy, as compared with those with ER (*p* < 0.001) (Figure 2).

**Figure 3:**
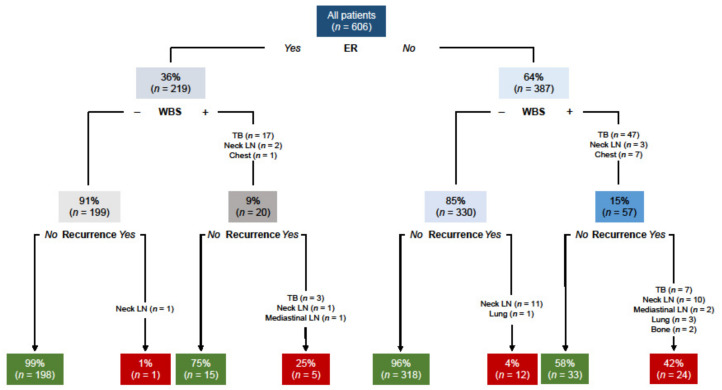
Outcome of patients according to 12-months response to therapy and diagnostic WBS findings. ER (excellent response), WBS (whole body scan), TB (thyroid bed), LN (lymph nodes).

To assess the role of diagnostic WBS, the outcome of patients according to 12-months response to therapy and WBS findings was evaluated (Figure 3). Among the 219 patients with ER according to serum Tg level and neck US findings, 20 (9%) had detectable foci of uptake at diagnostic WBS. During the subsequent follow-up, only five of these 20 (25%) patients had a structural recurrence with high serum Tg. One (1%) patient without detectable uptake at diagnostic WBS had recurrence in neck lymph nodes during follow-up. Among the 387 with no-ER patients, diagnostic WBS demonstrated uptake in 57 (15%) patients and 24 (42%) had recurrence during follow-up. On the other hand, among the 330 patients with no-ER and without detectable uptake at 12 months, diagnostic WBS recurrence occurred during follow-up in 12 (2%).

**Figure 4:**
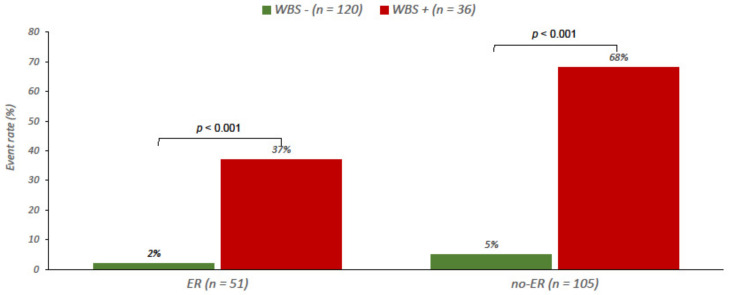
Event rate in patients at intermediate/high ATA risk and pre-therapy Tg ≥ 10 ng/mL, according to 12-months response to therapy by Tg and US and diagnostic WBS findings. ER (excellent response), WBS (whole body scan).

Finally, in 156 patients with intermediate/high ATA risk and pre-therapy Tg ≥10 ng/mL, the rate of events was higher in patients with abnormal diagnostic WBS in both ER (37% vs. 2%) and no-ER (68% vs. 5%) patients (both *p* < 0.001) (Figure 4).

## 4. Discussion

In this retrospective study we tested the incremental prognostic value of diagnostic WBS in the evaluation of response to ^131^I therapy in DTC patients treated with surgery and RAI. In our series, ATA risk classification and Tg level before RAI administration, as well as Tg level and US evaluation at 12 months, are the main predictors of outcome. The addition of diagnostic WBS at 12 months seems to be useless in low-risk patients considered in ER according to serum Tg level and neck US findings and it may still have a role in patients with intermediate/high ATA risk and pre-therapy Tg ≥ 10 ng/mL.

In patients with DTC an accurate risk stratification is essential in order to guide RAI treatment and follow-up. The AJCC/UICC TNM staging system has been developed to predict mortality according to histopathological findings and the presence of distant metastases [13,14]. However, TNM system is able to predict mortality but it revealed not ideal in assessing the risk of recurrence. The ATA initial risk stratification system is designed to predict the risk of recurrent or persistent disease in DTC patients and has been validated in different cohorts of DTC patients [6,9,15]. However, many DTC patients initially categorized as intermediate risk have varied clinical outcomes [16]. The addition of laboratories and imaging findings obtained during the first 12–24 months after treatment, including serum Tg and TgAb determinations or imaging procedures, can improve this initial risk assessment, and for this reason a dynamic approach has been proposed which considers different responses to treatment, such as ER, IR, BIR and SIR [6,15,17]. Integrative prognostic stratification systems, including molecular markers closely associated with non-RAI avidity, have also been proposed [18,19]. During follow-up, diagnostic WBS was not routinely performed to detect recurrent disease, due to its low sensitivity [20,21,22]. Undetectable levels of serum Tg is highly predictive of complete remission and the present data confirm that a diagnostic WBS may be avoided in these patients [20]. Furthermore, serum Tg may remain detectable during some months after initial treatment and subsequently disappear without any further treatment [5,23].

Recently, Known et al. [9] found that the proportion of patients without ER significantly increased when a radioiodine scan was added to the follow-up protocol. In our retrospective cohort, only eight patients with undetectable Tg and negative US were re-classified according to WBS results. The other 19 patients had persistent uptake in the thyroid bed, but this may only indicate the persistence of normal-non tumoral thyroid cells that have not been totally eradicated with initial treatment. These cells have been irradiated and will probably disappear with time and in fact none of these 19 patients experienced a structural recurrence and serum Tg remained undetectable.

Only one of the 219 patients classified as ER according to serum Tg level and neck US showed pathological uptake in the thoracic region and was re-classified as SIR. The presence of positive WBS despite negative Tg or TgAb values has been previously observed in some patients with cervical and mediastinal lymph nodes or small lung metastases [24,25]. Also, in the absence of SPECT-CT, the location of foci of uptake might be unreliable, as observed in one patient with uptake in the lateral neck that was not subsequently confirmed and that was probably thyroid bed uptake due to thyroid remnants.

Moreover, seven patients with detectable Tg levels, first classified as either IR for 2 or BIR for 5, were reclassified as SIR according to the discovery of abnormal uptake in the chest. These findings agree with current ATA guidelines [5] which recommend the use of diagnostic WBS only in patients with high or intermediate risk of persistent disease, in those patients with some evidence of disease such as detectable serum Tg level or suspicious findings at neck US [5]. In patients with detectable serum Tg during the first months after initial therapy, the slope of serum Tg levels over time might help to select patients for imaging (those with an increasing trend) and to follow-up those with a decreasing trend for whom the risk of recurrence is low.

## 5. Conclusions

Diagnostic WBS at 12 months has no significant incremental value in the identification of patients at higher risk of recurrence beyond Tg and US findings in low-risk patients with an excellent response to therapy. Diagnostic WBS may still have a role only in patients at intermediate-high ATA risk and in those with high pre-therapy Tg values.

## Figures and Tables

**Table 1:** Characteristics of the study population at the time of RAI therapy according to events.

Clinical Features	All (*n* = 606)	Event (*n* = 42)	No Event (*n* = 564)	*p*-Value
Age (years)	44 ± 14	47 ± 17	44 ± 14	0.12
Male gender, *n* (%)	106 (17)	12 (29)	94 (17)	0.05
ATA risk, *n* (%)	-	-	-	-
Low risk	153 (25)	2 (4)	151 (30)	<0.01
Intermediate risk	320 (53)	20 (48)	300 (53)	0.48
High risk	133 (22)	20 (48)	113 (20)	<0.001
Papillary type, *n* (%)	301 (50)	23 (55)	278 (49)	0.49
Follicular type, *n* (%)	109 (18)	5 (12)	104 (18)	0.29
Mixed, *n* (%)	194 (32)	13 (31)	181 (32)	0.88
Time interval surgery/therapy (days)	99 ± 46	88 ± 44	100 ± 46	0.10
Administered ^131^I activity (MBq)	3589 ± 922	3612 ± 814	3552 ± 925	0.69
Pre-therapy off Tg (ng/mL)	16 ± 39	43 ± 64	14 ± 35	<0.001
Pre-therapy off Tg ≥ 10 ng/mL, *n* (%)	198 (33)	22 (52)	92 (16)	<0.001
Post-therapy WBS, *n* (%)	-	-	-	-
Thyroid bed	594 (98)	42 (100)	552 (98)	0.34
Suspicious for lymph nodes	38 (6)	4 (10)	34 (6)	0.37

Data are presented as mean ± SD or number and percentage (%). Tg (thyroglobulin), WBS (whole body scan).

**Table 2:** Characteristics of the study population at 12 months evaluation according to events.

12-Months Evaluation	All (*n* = 606)	Event (*n* = 42)	No Event (*n* = 564)	*p*-Value
Post-therapy Tg (ng/mL)	4 ± 11	17 ± 29	4 ± 14	<0.001
Undetectable Tg/AbTg, *n* (%)	97 (31)	6 (16)	91 (32)	<0.05
Detectable Tg, *n* (%)	342 (56)	30 (71)	312 (55)	<0.01
Detectable AbTg, *n* (%)	38 (6)	5 (12)	33 (6)	0.12
Normal US, *n* (%)	572 (94)	35 (83)	537 (95)	<0.001
Abnormal US, *n* (%)	34 (6)	7 (17)	27 (5)	<0.001
Uptake at diagnostic WBS, *n* (%)	77 (13)	29 (70)	48 (9)	<0.001
Thyroid bed, *n* (%)	65 (11)	19 (45)	46 (8)	<0.001
Suspicious for lymph nodes, *n* (%)	2 (0.5)	2 (5)	2 (0.5)	<0.001
Extra-neck uptake, *n* (%)	8 (1)	8 (19)	0	-

Data are presented as mean ± SD or number and percentage (%). Tg (thyroglobulin), WBS (whole body scan), US (ultrasound).

**Table 3:** Characteristics of 42 patients with events at the time of recurrence.

ID	Pre Therapy		12 Months Evaluation				Recurrence			
	ATA Risk Category	Tg	Tg	US	Diagnostic WBS Uptake	Response to Therapy	Site	Months	Tg	Treatment
1	Intermediate	184	11	−	TB	BIR	TB	48	70	RAI
2	Intermediate	12	6	−	TB	BIR	TB	32	13	RAI
3	Intermediate	18	53	−	TB	BIR	TB	67	13	RAI
4	Intermediate	72	1.5	−	TB	BIR	TB	19	14	RAI
5	Low	32	1	+	TB	SIR	TB	18	29	RAI
6	High	73	1.4	+	TB	BIR	TB	90	11	RAI
7	Intermediate	36	7	+	TB	IR	TB	10	12	RAI
8	Intermediate	13	1.4	−	TB	IR	TB	17	9.1	RAI
9	Low	41	3	−	TB	IR	TB	17	59	RAI
10	Intermediate	24	0.1	−	TB	ER	TB	15	12	RAI
11	Intermediate	0.5	0.2	−	TB	ER	TB	17	28	RAI
12	Intermediate	51	2	+	TB	SIR	TB and LN	13	7.6	RAI
13	High	36	0.5	−	−	BIR	TB and LN	29	15	RAI
14	Intermediate	1	2	−	−	BIR	TB and LN	13	37	RAI
15	Intermediate	0.5	0.3	−	−	IR	TB and LN	25	8	RAI
16	High	320	11	−	−	IR	TB and LN	26	5.6	RAI
17	High	14	4.5	−	−	IR	Bone	73	330	RAI
18	High	1.1	1.4	−	Thorax	IR	Bone	19	49	RAI
19	High	19	2	−	Thorax	BIR	Lung	60	440	RAI
20	Intermediate	28	8	−	Thorax	BIR	Lung	11	83	RAI
21	High	102	5.2	−	Thorax	BIR	Lung	21	113	RAI
22	High	112	1.2	−	Thorax	IR	Lung	33	62	RAI
23	High	215	0.2	−	Mediastinum	ER	Mediastinal LN	17	30	RAI
24	High	2	4	−	Mediastinum	BIR	Mediastinal LN	16	8	RAI
25	High	67	45	−	Mediastinum	BIR	Paratracheal LN	25	9,7	RAI
26	Intermediate	14	0.7	−	LN	IR	TB and LN	18	4.2	Surgery + RAI
27	High	48	0.1	−	TB	IR	TB and LN	27	1.1	Surgery + RAI
28	High	0.8	0.2	−	LN	ER	LC LN	112	236	Surgery + RAI
29	Intermediate	24	0.1	−	−	ER	LC LN	12	6,1	Surgery + RAI
30	Intermediate	14	1.3	+	−	BIR	LC LN	56	4,7	Surgery + RAI
31	High	13	1.5	−	TB	BIR	LC LN	12	7,1	Surgery + RAI
32	High	64	1.2	+	LN	IR	LC LN	17	6,2	Surgery + RAI
33	High	6	5.1	−	−	BIR	LC LN	30	398	Surgery + RAI
34	High	37	20	−	TB	BIR	LC LN	12	196	Surgery + RAI
35	Intermediate	0.8	10	−	TB	BIR	LC LN	31	5,1	Surgery + RAI
36	High	0.5	2.5	−	−	IR	LC LN	55	2,2	Surgery + RAI
37	High	4	1.1	−	−	IR	LC LN	16	4,1	Surgery + RAI
38	High	3	0.6	−	−	IR	LC LN	32	9	Surgery + RAI
39	Intermediate	31	0.1	+	LN	IR	LC LN	19	10	Surgery + RAI
40	Intermediate	0.5	0.9	−	−	IR	LC LN	86	4,1	Surgery + RAI
41	Intermediate	7	8	−	−	BIR	LC LN	95	4	Surgery + RAI
42	Intermediate	51	0.2	−	TB	ER	LC LN	10	7	Surgery + RAI

Tg (thyroglobulin), TB (thyroid bed), LN (lymph nodes), LC (latero-cervical), RAI (radioactive iodine), US (ultrasound), ER (excellent response), IR (indeterminate response), BIR (biochemical incomplete response) SIR (structural incomplete response); +/−: presence/absence of thyroid residual tissue.

**Table 4:** Univariable and multivariable predictors of recurrence.

Variables	Univariable Analysis		Multivariable Analysis	
	Hazard Ratio (95% CI)	*p*-Value	Hazard Ratio (95% CI)	*p*-Value
ATA risk categories	-	-	-	-
Low (reference) (*n* = 153)	-	<0.001	-	<0.01
Intermediate (*n* = 320)	4.88 (1.14–20.89)	<0.05	5.99 (1.33–26.8)	<0.05
High (*n* = 133)	11.99 (2.80–51.29)	<0.01	14.6 (3.17–67.3)	<0.01
Pre-therapy Tg ≥ 10 ng/mL (*n* = 198)	4.87 (2.534–9.37)	<0.001	2.07 (2.25–7.78)	<0.001
12 months response to therapy (Tg + US)	-	-	-	-
ER (reference) (*n* = 219)	-	<0.01	-	-
IR (*n* = 182)	3.14 (1.20–8.00)	<0.05	3.24 (1.25–8.37)	<0.05
BIR (*n* = 203)	3.51 (1.40–8.78)	<0.01	2.88 (1.14–7.24)	<0.05
SIR (*n* = 2)	8.49 (2.85–25.30)	<0.001	17.4 (2.49–29.5)	<0.001

Tg (thyroglobulin), US (ultrasound), ER (excellent response), IR (indeterminate response) BIR (biochemical incomplete response), SIR (structural incomplete response).

## Data Availability

The data presented in this study are available on request from the corresponding author. The data are not publicly available due to privacy restrictions.
